# Risk assessment of trihalomethanes in drinking water with seasonal variation considerations

**DOI:** 10.1038/s41598-025-30481-9

**Published:** 2026-02-06

**Authors:** Gihan Hosny, Ashraf Elden, Samar Aborhyem

**Affiliations:** 1 Department of Environmental Studies (Division of Environmental Health), Institute of Graduate Studies and Research, Alexandria University, Alexandria, Egypt; 2 Department of Nutrition (Food Analysis), High Institute of Public Health, Alexandria University, EL-Hadrah, 165 EL-Horreya Avenue, Alexandria, 21561 Egypt

**Keywords:** Risk assessment, Trihalomethane, Hazard index, Drinking water, Cancer risk, Environmental sciences, Natural hazards

## Abstract

This study evaluated the concentrations of trihalomethanes (THMs) in Alexandria’s drinking water across seven districts during summer and winter, and assessed the associated cancer risks. THMs, byproducts of water chlorination, are known for their potential carcinogenicity. A total of 35 drinking water samples were analyzed using gas chromatography with an electron capture detector (GC-ECD) to determine the concentrations of chloroform (CF), bromodichloromethane (BDCM), dibromochloromethane (DBCM), and bromoform (BF). The results revealed significant seasonal variations. Region-2 recorded the highest summer concentration (150 µg/L), while Region-7 had the highest winter concentration (196 µg/L). Hazard index (HI) calculations indicated that chloroform contributed the most to non-carcinogenic risks, with Region-7 recording an HI of 0.5698 in winter, while Region-2 showed a summer HI of 0.4843. Carcinogenic risk assessments using the USEPA methodology showed lifetime cancer risks significantly exceeding the acceptable threshold of 1 × 10^−6^, with an annual average total cancer risk of 67.027 × 10^−6^. These outcomes highlight the critical need for Alexandria to adopt advanced water treatment methods, such as ozonation and UV disinfection, optimize chlorine dosing, and strengthen routine monitoring to ensure compliance with safety standards and protect public health to safer drinking water and better health outcomes for Alexandria’s residents.

## Introduction

Drinking water quality is a pressing public health concern due to its direct impact on human health. Ensuring water safety requires a comprehensive understanding of potential contaminants and their associated risks. Hazard identification serves as a critical first step in this process, involving the recognition of harmful substances, their sources, and potential exposure pathways. In the context of drinking water, one major category of hazardous compounds is disinfection by-products (DBPs), which form during the chlorination process. Among these, trihalomethanes (THMs) namely chloroform (CF), bromodichloromethane (BDCM), dibromochloromethane (DBCM), and bromoform (BF) are of particular concern due to their carcinogenic potential^1^. Chemically, THMs form through halogenation reactions between chlorine or bromine and organic precursors such as humic and fulvic acids. The formation of THMs can be influenced by various factors, including the concentration of natural organic matter, chlorine dose, and water pH. Additionally, the presence of bromide in water can significantly enhance THM formation, particularly brominated THMs like BDCM and DBCM Hypothetically, increased temperatures and altered water conditions during different seasons may influence the reactivity of chlorine and organic matter, thereby affecting THM concentrations^1^.

The occurrence of Natural Organic Matter (NOM) is ubiquitous, not only in soil and sediments but also in natural water resources. NOM is primarily comprised of humic acid (HA) and fulvic acid (FA), mainly formed during the decomposition of organic matter and algal metabolic activity. The properties of NOM depend upon its source of origin and biodegradability of organic matter. This can be classified based on polarity and hydrophobicity. The nonpolar hydrophobic fraction of NOM includes aromatic amines, hydrocarbons, and phenolic compounds, whereas the major constituent of the polar hydrophilic fraction contains carboxylic acid and aliphatic carbons. These phenolic and carboxylic functional groups of NOM instantly react with chlorine and provoke the formation of Trihalomethane (THMs) compounds, i.e., chloroform (CF), bromodichloromethane (BDCM), dibromochloromethane (DBCM), and bromoform (BF) in drinking water.^2^

Kumari and Gupta^3^ highlighted that exposure to THMs in drinking water has been linked to various health risks, including carcinogenic and non-carcinogenic effects, necessitating rigorous monitoring and control measures to mitigate human exposure. This seasonal variation is hypothesized to result from higher temperatures accelerating chemical reactions involved in THM formation, coupled with potential changes in water quality parameters and disinfection practices. Internationally, THM levels exhibit a broad range of concentrations. European countries have made significant progress in reducing THM levels through improved water treatment technologies and infrastructure upgrades^4^. France has optimized chlorine dosing to minimize disinfection by-products, while Italy’s use of chlorine dioxide has reduced THM levels but introduced other by-products such as chlorites (Shi et al., 2024). Other countries use alternative disinfection methods like ozone or UV radiation to mitigate THM formation^5^.

Mahato and Gupta^6^ demonstrated that advanced adsorption techniques using cerium oxide nanoparticles (CONPs) show exceptional efficiency in removing natural organic matter (NOM), which is a key precursor to THM formation, indicating potential for improved water treatment strategies. Furthermore, Mahato and Gupta^7^ highlighted the potential of predictive modeling techniques, such as artificial intelligence and multivariate regression, in estimating THM concentrations in drinking water, which can enhance risk assessment and inform regulatory decision-making.

The novel contribution of this research lies in it being the first attempt to assess the levels of THMs in Alexandria’s drinking water across various districts and seasons, evaluate the associated non-carcinogenic and carcinogenic risks, and compare these results with international data. By analyzing the seasonal effects on THM levels and the potential chemical mechanisms driving these variations, unlike previous studies that often focus on singular assessments, this investigation comprehensively examines temporal fluctuations in THM levels and their health risks, offering actionable recommendations for more adaptive water treatment strategies tailored to seasonal changes.

## Material and methods

**Fig. 1:**
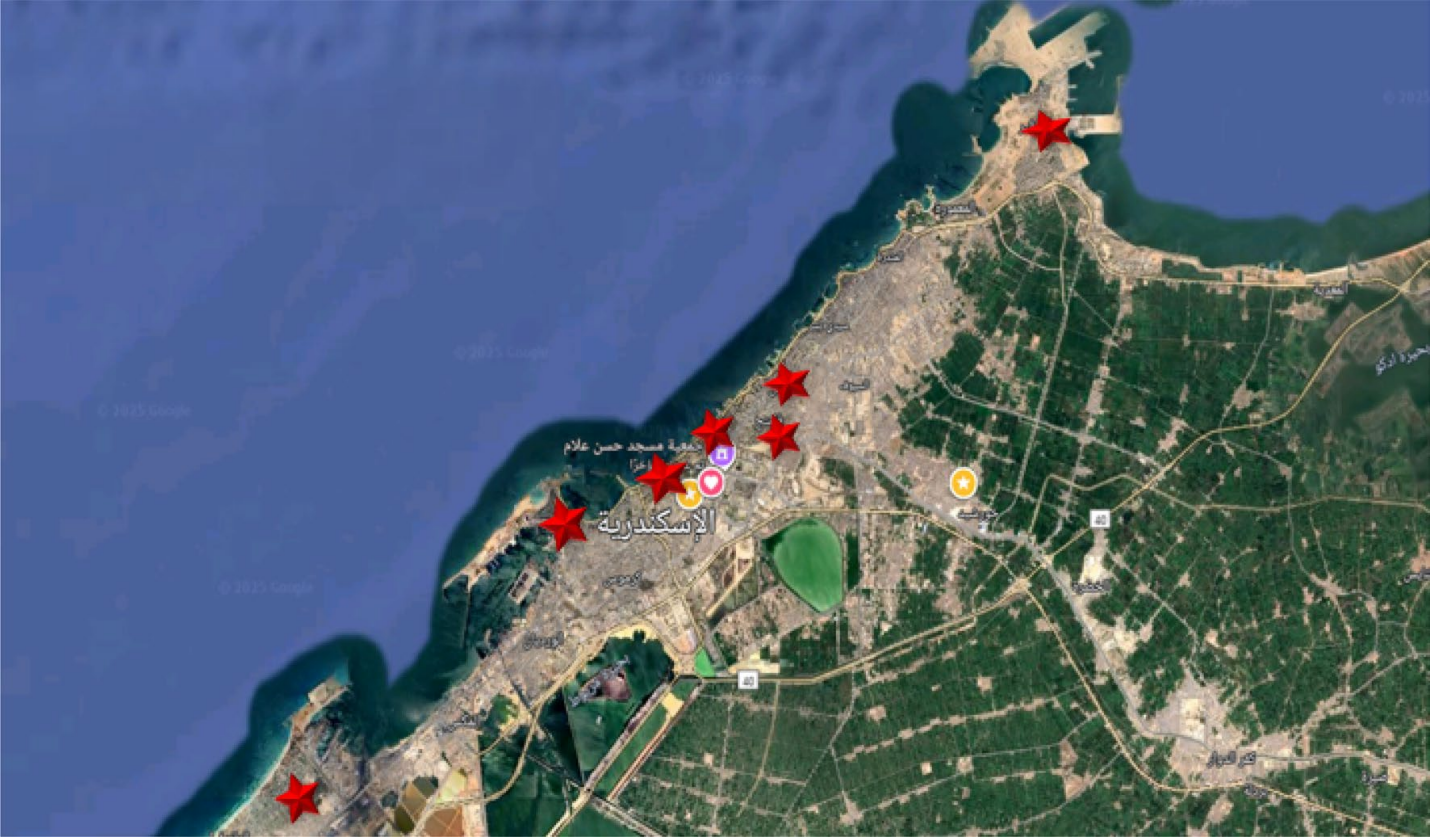
Map showing the sampling locations (indicated by red stars) used for water collection and analysis.

A cross-sectional study was conducted to assess water quality in Alexandria by collecting drinking tap water samples from seven randomly selected districts: Region-1, Region-2, Region-3, Region-4, Region-5, Region-6, and Region-7 as mention in Fig. 1. These districts were chosen to ensure diverse geographic and socio-economic representation, capturing a wide range of environmental and infrastructural conditions across Alexandria. While the city is divided into nine districts, selecting seven allowed us to maintain a balance between practicality and comprehensive coverage. The districts selected represent both coastal and inland areas, ensuring that variations in water quality due to environmental factors were considered. Moreover, the districts reflect different socio-economic levels, which can influence water infrastructure and quality, further enriching the representativeness of the study.

Drinking water samples were collected from the 1^st^ floor garage of residential buildings, with a total of 35 samples gathered between August and October 2020. Each district contributed three samples collected at different times of the day. To effectively collect five water samples throughout the day, a strategic sampling schedule was implemented to capture variations in water quality. The first sample was taken in the early morning (e.g., 6:00 AM–7:00 AM) before heavy household water use begins, reflecting potential stagnation issues. The second sample was collected mid-morning (e.g., 10:00 AM–11:00 AM) when water usage starts to increase, which may reveal changes due to rising demand. The third sample was gathered around midday (e.g., 1:00 PM–2:00 PM) during a period of moderate usage to assess the system’s performance. The fourth sample was taken in the afternoon (e.g., 4:00 PM–5:00 PM) when usage peaks again, capturing how the system responds to high demand. Finally, the fifth sample was collected in the evening (e.g., 8:00 PM–9:00 PM), after peak usage, to evaluate water quality as demand begins to decline. Throughout the sampling process, it is essential to maintain consistency in timing, document all relevant details, and use sterilized containers to avoid contamination, ensuring that the collected data is representative and reliable. The mean ambient temperature during this period ranged from 25 °C to 33 °C, offering insight into the impact of warmer months. To examine seasonal variations, the same districts were sampled again between January and March 2021, when the mean ambient temperature ranged from 10 °C to 18 °C, allowing a comparison of water quality in both summer and winter seasons. This method of sampling across different times of day and seasons ensured that the data collected was not only representative but also capable of identifying any temporal variations in water quality.

### Rigorous application of standardized methods for accurate quantification of trihalomethanes and chlorinated organic compounds: ensuring precision and data integrity

In this study, the Standard Methods for the Examination of Water and Wastewater, 24^th^ Edition were applied to ensure the precise determination of four key trihalomethanes (THMs)–chloroform (CF), bromodichloromethane (BDCM), dibromochloromethane (DBCM), and bromoform (BF)^8^. A key aspect of this study’s strength is the rigorous adherence to quality control (QC) practices, which are integral to ensuring the reliability and accuracy of the data. Precision and bias data were calculated for each compound using a 5%-Phenyl-methylpolysiloxane nonpolar column, known for its low bleed and high-temperature tolerance. The recovery rates for all compounds fall within the accepted range of 80–120%, with low relative standard deviations (RSD) indicating a high degree of precision across replicates. For instance, chloroform (CHCl₃) demonstrated a recovery rate of 95.6% with a 2.4% RSD, and carbon tetrachloride (CCl₄) showed 100.8% recovery with a 1.2% RSD, confirming the reliability of the method for quantifying these compounds.

In addition, the calibration and verification steps were performed using internal standards; 1, 2 dibromopropane that have a baseline resolution to separate it from constituents of interest and any interference. The internal standard yielded a mean recovery rate of 100.8%, with an RSD of 3.3%, further validating the robustness of the analytical process. These data provide confidence in the measured values and their reproducibility, demonstrating that the uncertainty associated with the measurements has been meticulously calculated and controlled.

The rigorous application of these standardized methods, along with stringent quality control measures, supports the overall precision and reliability of the results, minimizing uncertainty. The study exemplifies a robust application of standardized analytical methods, providing full transparency regarding QA/QC measures to ensure the accuracy and reliability of the data. The precision and recovery metrics demonstrate that the data generated is not only representative but also repeatable. This rigorous approach is essential for assessing water quality and ensuring compliance with regulatory standards.

### Quality control (QC) check standards

**Fig. 2:**
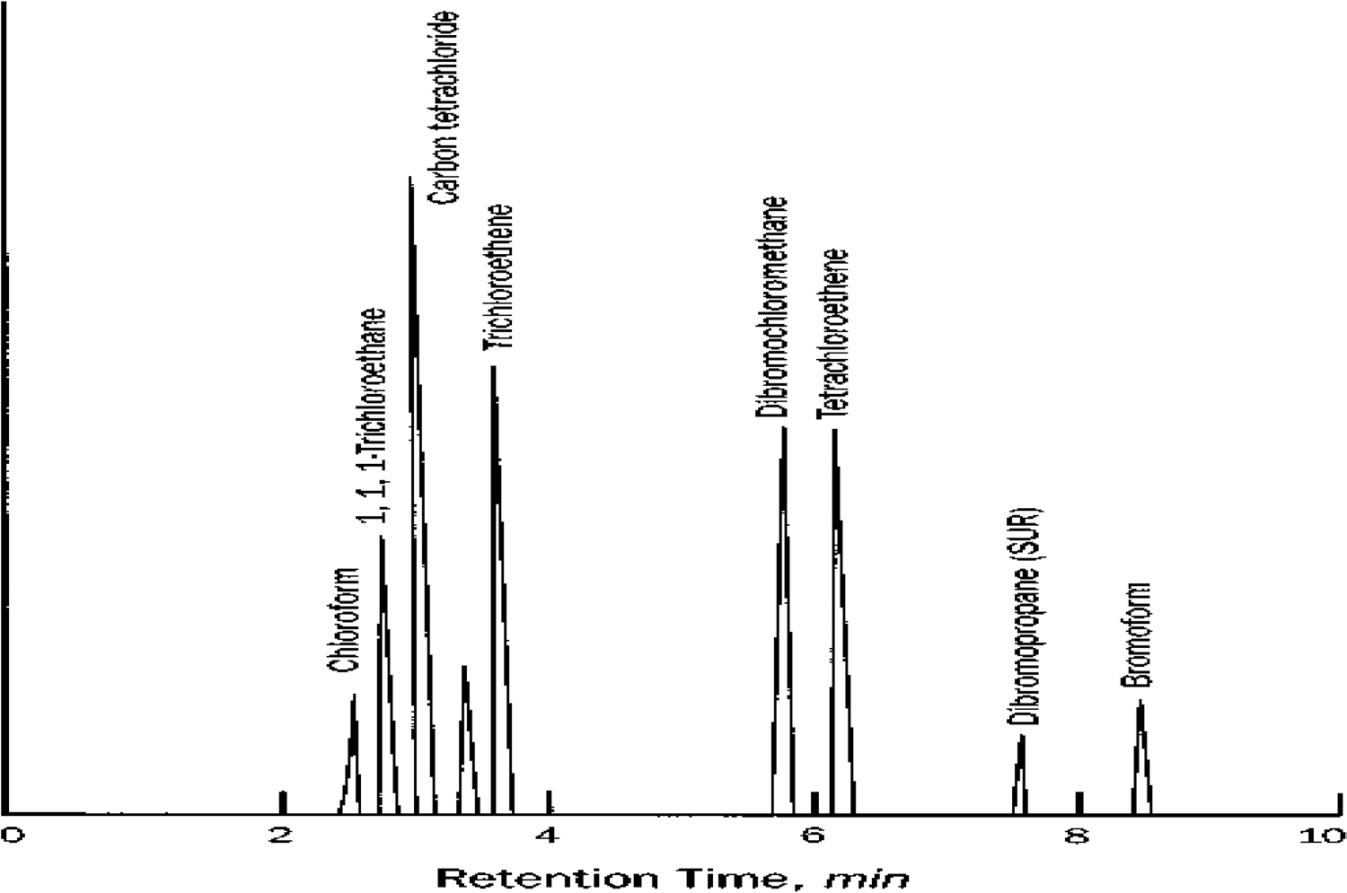
Chromatogram for THMs and chlorinated organic solvents.

A calibration curve for each ingredient was created using at least three concentrations, but ideally five to seven. For each chemical, a concentration in methanol was got from a proficiency-testing materials vendor. Stock standards were prepared separately from tidy materials used for calibration standards. A mixed secondary dilution standard was prepared with each component, followed by an aqueous QC check standard with a concentration close to the midlevel calibration standard^9^. QC standards were preferably obtained from a separate source and prepared independently from calibration standards. QC check standards were analyzed as though they were samples at a frequency of 5% (every 20 samples) and at the end of the analytical sequence. Results were compared to known concentration of the check standard and percent recovery was calculated. Percent recovery nominally should be between 80 and 120%. Mean recovery control charts of QC check standards results were developed and 99% confidence limits were used to accept or reject the ongoing calibration. Figure 2 illustrated the chromatogram for THMs and chlorinated organic solvents. Concentration was 50 µg/L for each compound; primary column DB-5.

### Quality control

The quality control practices considered to be an integral part of each method are summarized in Table 1. A minimum program of quality control consists of an initial demonstration of proficiency for each analyst and each instrument system and an ongoing program of quality control analysis. Record initial quality by documenting initial performance relative to published performance criteria. Maintain records of performance by comparing ongoing quality control checks to performance criteria and objectives for data quality^9^. The Limit of Detection (LOD) and Limit of Quantification (LOQ) for all THM compounds were determined following standard validation protocols. LOD was calculated based on a signal-to-noise ratio of 3:1, while LOQ was based on a 10:1 ratio.

**Table 1:** Precision and bias data for the chlorinated organic solvent method, db5 column.

Compound	Added Amount	A	B	C	D	E	F	G	H	Bias %*Recovery*	Precision %*RSD*
Chloroform (CHCl_3_)	20.0	18.7	18.6	19.4	19.5	19.2	18.5	19.5	19.7	95.6	2.4
Bromodichloromethane (BDCM)	20.0	19.8	20.2	20.7	20.8	20.3	19.7	20.6	20.5	101.6	2.1
Dibromochloromethane (DBCM)	20.0	18.7	19.4	20.0	20.2	19.7	18.7	20.1	20.6	98.3	3.6
Bromoform (CHBr_3_)	20.0	17.4	18.5	18.7	19.2	19.3	17.9	18.8	19.8	93.5	4.1
Trichloroethane (TCA)	20.0	18.5	18.8	19.7	19.9	19.8	18.5	20.1	20.4	97.3	3.8
Carbontetrachloride (CCl4)	20.0	20.1	20.4	20.0	20.2	20.6	20.0	20.1	20.0	100.8	1.2
Trichloroethene (TCE)	20.0	17.9	18.3	18.9	19.2	19.1	17.9	19.2	19.6	93.8	3.4
Tetrachloroethene (PCE)	20.0	19.8	20.4	20.6	20.9	20.7	19.7	20.7	20.7	102.2	2.2
Internalstandard	100.0	99.0	95.0	100.0	102.0	101.0	99.0	105.0	105.0	100.8	3.3

LOD: 0.5 µg/L, LOQ: 1.5 µg/L.

### Sampling procedures

Sodium thiosulfate solution was applied to the amber bottles to remove any residual chlorine and prevent the development of THM. Each one of glassware was cleaned with phosphate-free detergent and then rinsed with milli-Q ultrapure water and acetone (HPLC grade). After that, it was baked at 150 °C for 2 h and then chilled at room temperature till sampling the water samples in 40 mL amber glass bottles, which were filled without air bubbles^10^.

### Sample preparation and analysis

Samples were made by mixing 10 ml of water with 2 ml of pentane for 2 min in a 25 ml separating funnel. The upper phase was collected into 2 ml vials with screw covers and Polytetrafluoroethylene (PTFE) (Teflon septum) after 3 min of phase separation. Gas chromatography- Electron capture detector (GC–ECD), Varian, Model CP-3800) was used to measure Tri-Halogenated Methane compounds (THMs) levels. Extraction and analysis time per sample ranges from 10 to 30 min. Mass spectrometry was used to gather further confirmation evidence for confirmatory studies. Under identical conditions, standards are introduced to organic-free water, and samples are extracted and evaluated in the same way. This step is required to ensure that the simplified extraction approach achieves 100% extraction efficiency^11^. A capillary column DB-5 (J&W Scientific Inc/Agilent Technologies, 30 m length, and 0.25 mm capillary size) was used for chromatographic separation. The temperature schedule for the GC oven was as follows: 40 °C for 2 min, then 10 °C each minute until 150 °C. Each experiment utilized a carrier gas (N_2_) at a flow rate of 0.8 ml min-1 and a split ratio of 1:10. In the capillary column, 2 µl of each sample was injected. Samples were analyzed at the central laboratory of Alexandria Water Company at Alnozha airport. They used to analyze samples by "6232 b. Liquid Extraction Gas Chromatographic Method^11^.

Standard Methods for the Examination of Water and Wastewater, 24th Edition is applicable to determine four trihalomethanes (THMs; i.e., chloroform (CF), bromodichloromethane (BDCM), dibromochloromethane (DBCM), and bromoform (BF)). Calibration standards for bromoform (CHBr3), bromodichloromethane (BDCM), dibromochloromethane (DBCM), chloroform (CHCl3), 1,1,1-trichloroethane (TCA), tetrachloroethene (PCE), trichloroethene (TCE), and carbon tetrachloride (CCl_4_). The quality control practices considered to be an integral part of each method were applied to ensure the precision of data and calculate uncertainty as well as the repeatability of the obtained data^8^.

### Risk assessment calculation

Exposure routes to tri-halogenated methane compounds (THMs) from tap water are ingestion, inhalation, and dermal absorption. The risk assessment process includes four components: data collection and evaluation, exposure assessment, toxicity assessment, and risk characterization. Results are then integrated and compared to the estimated uptake with appropriate toxicological values to determine the likelihood of adverse effects in potentially exposed populations^12^.

In this study, two approved risk assessment models were adopted: World Health Organization (WHO) index for additive toxicity (I_WHO_), and the USEPA-approved Risk Assistant model adopted by many researchers^13–17^. The WHO Index for Additive Toxicity, I_WHO_, for THMs is an overall guideline value to estimate the toxic (developmental and non-carcinogenic) risk associated with chlorinated drinking water. The ${\mathrm{I}}_{\mathrm{WHO}}$ value should be ≤ 1 for compliance with WHO guidelines and is calculated as follows:

$${\mathrm{I}}_{\mathrm{WHO}}=\frac{{C}_{CF}}{{GV}_{CF}}+\frac{{C}_{BDCM}}{{GV}_{BDCM}}+\frac{{C}_{DBCM}}{{GV}_{DBCM}}+\frac{{C}_{BF}}{{GV}_{BF}}\le 1$$

where C is the concentration of each THM in this study, and GV is the WHO guideline values that have been established. The GV for CF is 200, BDCM 60, DBCM 100, and BF 100, all in μg/l^18^.

The USEPA Risk Assessment Model is capable of assessing both toxic and non-carcinogenic hazards, and toxicological risks, represented as the hazard quotient (HQ), are computed using the following formula:

$$\text{HQ }= \frac{\text{Total amount ingested}}{\text{Body weight}\times \text{ Exposure time} \times \text{ Reference dose}(\mathrm{RFD}) }=\frac{\mathrm{LaDD}}{\mathrm{RFD}}$$

where RfD is the Reference Dose and LADD is the Life Average Daily Dose which is calculated for three pathways

$${\mathrm{LADD}}_{\mathrm{oral}}= \frac{\text{Total amount ingested}}{\mathrm{Bodyweight}\times \mathrm{lifetime}}=\frac{(\mathrm{Conc}.\text{ THM in water}\times \mathrm{ER}\times \mathrm{EF}\times \mathrm{ED})}{(\mathrm{BW}\times \mathrm{AT})}$$

$${\mathrm{LADD}}_{\mathrm{dermal}}=\frac{(\mathrm{Conc}.\text{ THM in water}\times \mathrm{SA}\times \mathrm{PC}\times \mathrm{ET}\times \mathrm{EF}\times \mathrm{ED})}{(\mathrm{BW}\times \mathrm{AT})}$$

$${\mathrm{LADD}}_{\mathrm{inhalation}}=\frac{(\mathrm{Conc}.\text{ CF in water}\times \mathrm{AA}\times \mathrm{VF}\times \mathrm{ET}\times \mathrm{EF}\times \mathrm{ED})}{(\mathrm{BW}\times \mathrm{AT})}$$

Table 2 showed the input factors and abbreviations for exposure assessment^9^. Toxicological studies of exposure that show a critical impact are used to calculate reference dosages. They are accessible in the integrated risk information system database and are represented in mg/kg/day^19^. The overall quantity of chemicals consumed is determined by several common or population-specific exposure variables, including chemical concentration in local waterways, water consumption rate, frequency of exposure, and length of exposure. Estimates of body weight and exposure duration are also required to determine hazard quotient (HQ). Based on the exposure variables, HQs in the Alexandria population were calculated using the USEPA method.

**Table 2:** Input factors and abbreviations for exposure assessment (Semerjian & Dennis, 2007).

Input parameter	Unit	Value	References
THM Conc. in water (C)	mg/l	See tables	This study
Exposure rate (ER)	L/day	2.0	US EPA (1997)
Exposure frequency (EF)	days/ year	365	Lee et al. (2004)
Exposure duration (ED)	year	70	US EPA (1997)
Average exposure time (AT)	days/year	70 × 365	Lee et al. (2004)
Body weight (BW)	kg	70	Lee et al. (2004)
Surface area (SA)	M^2^	1.8	US EPA (1997)
Exposure time (ET)	min/day	35 min	RAIS (2009)
Aspirated air (AA)	m^3^ per day	20 = 0.83333 m^3^/hr	Semerjian and Dennis (2007)
Volatilization factor for chloroform (VF)	L/ m^3^	0.5	Semerjian and Dennis (2007)
Permeability coefficient (PC)	cm/h	0.00683 (CF) 0.00402(BDCM) 0.00289(DBCM) 0.00235(BF)	RAIS (2009)

If the HQ value is less than 1, the exposed local population is deemed safe; if the HQ value is equal to or more than 1, it is considered unsafe for human health, and appropriate actions and preventive measures should be performed. The hazard index is the sum of the hazed quotients for all THMs which was calculated.

$$\mathrm{HI}=\sum \mathrm{HQ}={\mathrm{HQ}}_{\mathrm{CF}}+{\mathrm{HQ}}_{\mathrm{BDCM}}+{\mathrm{HQ}}_{\mathrm{DBCM}}+{\mathrm{HQ}}_{\mathrm{BF}}$$

The total exposure hazard index is estimated by adding the hazard index (HI) of each exposure pathway together and in this case are ingestion, dermal, and inhalation.

$$\begin{aligned} Total\;Exposure\;Hazard\;Index = & Hazard\;Index\;\left( {ingestion} \right) + Hazard\;Index\;\left( {dermal} \right) \\ & + Hazard\;Index\;\left( {inhalation} \right) \\ \end{aligned}$$

The reference doses (RfD), cancer group classifications for the THM components, and cancer slope factors (CSF) for oral, dermal, and inhalation used for THM via different routes^19^. In addition to toxic risks, carcinogenic risks of exposure to surveyed THM levels were calculated using the USEPA methodology. Carcinogenic compounds differ from toxic compounds in that there is no lower limit for the existence of risk. Thus, carcinogen risk assessment models are generally based on the premise that risk is proportional to total lifetime dose, and the exposure metric used for carcinogenic risk assessment is the Lifetime Average Daily Dose (LADD). The LADD is typically used in conjunction with the Cancer Slope Factor (CSF) to calculate individual excess cancer risk.

The carcinogenic slope factors (CSF) and reference doses (RfD) represent crucial toxicological values for assessing the health risks of trihalomethanes (THMs) in drinking water. Here’s a summary of the values for each THM component^19^: Carcinogenic Slope Factors (CSF) (mg/kg/day).

- Chloroform (CF): 3.1 × 10^−2^ (oral/dermal)
- Bromodichloromethane (BDCM): 6.2 × 10^−2^ (oral/dermal)
- Dibromochloromethane (DBCM): 8.4 × 10^−2^ (oral/dermal)
- Bromoform (BF): 7.9 × 10^−2^ (oral/dermal)

Reference Doses (RfD) (mg/kg/day).

Oral Contact:

- CF: 0.01
- BDCM, DBCM, BF: 0.02

Dermal Contact:

- CF, BDCM, DBCM: 0.02
- BF: 0.012
  $$THM\;carcinogenic\;risk\;of\;oral\;route = LADD\;oral \times CSF\;oral$$
  $$THM\; \, carcinogenic\;risk\;of\;dermal\;absorption = LADD\;dermal \times CSF\; \, ora$$
  $$THM\;carcinogenic\;risk\;of\;inhalation = LADD\;inhalation \times CSF\;inhalation$$

Where

$${\mathrm{LADD}}_{\mathrm{oral}}= \frac{\text{Total amount in gested}}{\text{Body weight}\times \text{life time}}=\frac{(\mathrm{Conc}.\text{ THM in water} \times \mathrm{ER} \times \mathrm{EF}\times \mathrm{ED})}{(\mathrm{BW}\times \mathrm{AT})}$$

$${\mathrm{LADD}}_{\mathrm{dermal}}=\frac{(\mathrm{Conc}.\text{ THM in water}\times \mathrm{SA}\times \mathrm{PC}\times \mathrm{ET}\times \mathrm{EF}\times \mathrm{ED})}{(\mathrm{BW}\times \mathrm{AT})}$$

$${\mathrm{LADD}}_{\mathrm{inhalation}}=\frac{(\mathrm{Conc}.\text{ CF in water}\times \mathrm{AA}\times \mathrm{VF}\times \mathrm{ET}\times \mathrm{EF}\times \mathrm{ED})}{(\mathrm{BW}\times \mathrm{AT})}$$

## Results and discussion

### THMs levels (μg/l)

The use of chlorine to disinfect drinking water is linked to the formation of THMs and other halogenated organics^20^. THMs are chemicals that are thought to be carcinogenic and mutagenic^21^. Villanueva, Evlampidou^22^ Stated that the maximum contamination level (MCL) of 100 μg/l and a chloroform acceptable level of 30 μg/l for drinking water. THMs (CHCl_3_, CHCl_2_Br, CHClBr_2_, and CHBr_3_) were among the most common pollutants identified in water samples, according to preliminary research. Only three samples contained 1, 2-dichlorobenzene at relatively low concentrations (39–78 μg/l). Total THM residues were found in all of the collected water samples except water collected from region 5 in summer, and region 3 in winter season.

The data presented in Table 3 reveal a clear seasonal variation in total trihalomethane (THM) concentrations, with markedly higher levels observed during the summer months across the majority of sampling sites. For example, Region-2 recorded a significant increase in total THMs during summer (150 ± 0.26 μg/L) compared to winter (53 ± 0.00 μg/L), while Region-3 exhibited detectable THMs only during the summer (35 ± 0.09 μg/L), with concentrations falling below detection limits in winter. Notably, Region-7 recorded the highest winter THM level (196 ± 0.10 μg/L), surpassing its summer value (27 ± 0.09 μg/L), suggesting the influence of site-specific factors on THM formation as well as suggesting that factors other than temperature such as variations in organic precursor availability, chlorine dose, and hydraulic conditions may also influence THM formation. These findings indicate that THM generation is governed by a complex interplay of physicochemical and environmental parameters rather than temperature alone.

The paired t-test analysis demonstrated statistically significant seasonal variations (p < 0.001) in the concentrations of individual and total trihalomethanes (THMs) across most sampling regions (Table 3). This temperature-dependent increase in THM formation suggests that local factors such as treatment practices, water stagnation, and distribution infrastructure play significant roles in THM formation, in addition to temperature-driven chemical kinetics. Collectively, these results underscore the importance of continuous seasonal monitoring and the application of optimized disinfection strategies to minimize THM accumulation and ensure safe drinking water quality throughout the year. The general trend supports the hypothesis that elevated temperatures in summer enhance THM formation, likely due to increased reaction kinetics between chlorine-based disinfectants and natural organic matter. Nonetheless, exceptions such as the elevated winter concentrations in Regions 5 and 7 highlight the need for further investigation into local water treatment processes and distribution system dynamics. These findings clarified the importance of continuous seasonal monitoring and tailored risk assessments to ensure the safety and regulatory compliance of drinking water with respect to THM contamination.

The results of the present study were generally within the EPA’s allowed levels, but greater than those suggested by the European Economic Community in different nations. However, it’s important to mention that the acceptable limits for THMs in drinking water vary by country. For instance, the United States has set a limit value of 80 μg/L for Total Trihalomethanes (TTHMs), while the European Union has a THM limit of 100 μg/L^23^. Total THMs in drinking water from different nations illustrated in the following Table.

**Table 3:** Mean values of individual and total THMs (μg/l) in water samples collected in summer and winter seasons.

		Region 1	Region 2	Region 3	Region 4	Region 5	Region 6	Region 7	LOD	LOQ
CHCL3	Summer	8.02^#^ ± 0.04	124.0^#^ ± 0.12	17.10^#^ ± 0.20	8.18^#^ ± 0.33	0.0 ± 0.0	14.94^#^ ± 0.09	12.08^#^ ± 0.51	0.5	1.5
	Winter	4.06^#^ ± 0.25	43.90^#^ ± 0.52	0.0 ± 0.0	36.0^#^ ± 0.80	63.01^#^ ± 0.03	21.02^#^ ± 0.19	180.0^#^ ± 0.90		
	t	31.757*	369.474*	191.184*	62.458*	4523.711*	49.502*	301.072*		
	p	< 0.001*	< 0.001*	< 0.001*	< 0.001*	< 0.001*	< 0.001*	< 0.001*		
CHCL2BR	Summer	7.95^#^ ± 0.25	19.0^#^ ± 0.43	10.99^#^ ± 0.57	7.0^#^ ± 0.02	0.0 ± 0.0	13.0^#^ ± 0.0	9.01^#^ ± 0.34	0.2	0.60.1
	Winter	0.0 ± 0.0	4.98^#^ ± 0.23	0.0 ± 0.0	0.0 ± 0.0	4.94^#^ ± 0.25	0.0 ± 0.0	12.01^#^ ± 0.02		
	t	71.050*	70.839*	43.093*	737.865*	43.315*	–	19.637*		
	p	< 0.001*	< 0.001*	< 0.001*	< 0.001*	< 0.001*	–	< 0.001*		
CHCLBR2	Summer	6.03^#^ ± 0.12	7.01^#^ ± 0.02	7.04^#^ ± 0.25	4.99^#^ ± 0.19	0.0 ± 0.0	8.02^#^ ± 0.20	6.01^#^ ± 0.13	0.1	0.3
	Winter	0.0 ± 0.0	4.0^#^ ± 0.13	0.0 ± 0.0	5.01^#^ ± 0.05	5.09^#^ ± 0.32	4.01^#^ ± 0.03	4.04^#^ ± 0.12		
	T	111.784*	57.927*	62.741*	**0.270**	35.041*	44.884*	48.100*		
	P	< 0.001*	< 0.001*	< 0.001*	**0.800**	< 0.001*	< 0.001*	< 0.001*		
THMs	Summer	22.0^#^ ± 0.22	150^#^ ± 0.26	35.0^#^ ± 0.09	20^#^ ± 0.31	0.0 ± 0.0	36^#^ ± 0.02	27 ^#^ ± 0.09	0.5	15
	Winter	4.01^#^ ± .01	53.0 ± 0.0	0.0 ± 0.0	41^#^ ± 0.03	73^#^ ± 0.10	25^#^ ± 0.42	196^#^ ± 0.1		
	T	179.575*	787.980*	802.065*	160.956*	1635.390*	56.575*	2579.097*		
	P	< 0.001*	< 0.001*	< 0.001*	< 0.001*	< 0.001*	< 0.001*	< 0.001*		

5 replica in each subgroup. Data was expressed using Mean ± SD. SD: Standard deviation, t: Paired t-test. p: p value for comparing between Summer and Winter in each Region. *Statistically significant at p ≤ 0.05. ^#^Significant with the reference value using one sample t-test. 0.00 Means ND: Not detected lower than the limit of detection.

**Table:** 

Country	THM Concentration (µg/L)
Japan	12.5 – 37.5 (Mean: 15.8)
Thailand	Mean: 44.9
Sweden	0.2 – 25
Germany	0.5
Russia	215
China	3
Bangladesh	439.2

The developed country has partially succeeded in reducing THM concentration in drinking water, whereas significant steps are needed in developing countries to reduce the existing high THM concentration. The concentration of THMs in water varies among these countries because of the different water sources, water quality, environmental conditions, and efficiency of water treatment technologies. A meaningful relationship has been observed between the properties of water and the THM formation^4^.

Flocculation, sedimentation, and fast sand filtration are all common water treatment methods. Chlorine is added to raw water (pre-chlorination) and filtered water as a disinfectant (post-chlorination). Water pre-chlorination, together with longer contact time in settling basins and clean wells plays a role in the development of THMs. They also suggested that sand filters might be a source of THM precursors if residual microbial life releases too many compounds. Other than chloroform, the carcinogenic consequences of THMs are unknown. They are, however, known to be more active in the Ames Salmonella mutagenesis test than chloroform^24^. Because dichlorobromomethane is structurally identical to chloroform, an MCL of 30 g/l will be considered for that THM species in this investigation. There are clear indications that the EPA’s THM limits may be reduced^25^

In addition to the currently applied models for estimating health risks associated with trihalomethanes (THMs), the study by Samayamanthula et al.^26^ introduces a comprehensive health risk index specifically developed for evaluating THM exposure in indoor swimming pool environments in Kuwait. Although the study was conducted in a different setting, the methodological framework it proposes offers valuable insights that could inform future efforts to assess and compare THM-related health risks across various water use scenarios. The structured approach outlined in their work provides a useful benchmark for expanding risk assessment models beyond drinking water systems and highlights the importance of contextual factors in evaluating human health exposure to disinfection by-products^26^.

### Non-carcinogenic risk for THM

#### The WHO Index for the toxicity approach

Calculated I_WHO_ values for network THM levels recorded for individual locations of Alexandria distribution are summarized in Table 4. The WHO Index (I_WHO_) for toxicity is a critical tool for assessing the potential health risks posed by trihalomethanes (THMs) in drinking water. The calculated I_WHO_ values for different sampling sites within the Alexandria distribution network reveal seasonal variations, with higher toxicity levels generally observed in certain locations during winter, such as the Region-7 water treatment plant, where the I_WHO_ reached up to 1.14. Conversely, Region-2 exhibited the highest I_WHO_ value during the summer at 1.005. These fluctuations indicating the importance of continuous monitoring.

**Table 4:** I_WHO_ values for THM levels were recorded for individual locations in Alexandria.

Sampling site	I_WHO_	
	Summer	Winter
Calculated I_WHO_	0.201–1.005	0.021–1.410
Region-1	0.23	0.021
Region-2	1.005	0.341
Region-3	0.338	ND
Region-4	0.201	0.233
Region-5	ND	0.442
Region-6	0.37	0.145
Region-7	0.27	1.140

#### Life average daily dose (LADD)

Life average daily dose (LADD) value was calculated considering the type of exposure The Life Average Daily Dose (LADD) for trihalomethanes (THMs) during the summer season highlights the varying levels of exposure through oral, dermal, and inhalation routes. Region-2 exhibited the highest total LADD, with chloroform (CF) contributing significantly across all exposure routes, particularly oral ingestion (4.05 μg/kg/day). The cumulative LADD for all THMs at Region-2 reached 5.242 μg/kg/day, which is considerably higher than the other locations.

In contrast, bromoform (BF) was not detected in any of the sites. Different areas within Alexandria have varying levels of THM exposure risks, with Region-2 being the most affected during the summer. The data showed the importance of localized risk assessments to better understand and mitigate potential health impacts. The Life Average Daily Dose (LADD) for trihalomethanes (THMs) during the winter season in Alexandria indicates that chloroform (CF) is the predominant THM contributing to the total exposure. Region-7 recorded the highest total LADD for CF (6.878 μg/kg/day) with significant contributions from all exposure routes, particularly oral ingestion.

In comparison, bromodichloromethane (BDCM) and dibromochloromethane (DBCM) were present at much lower levels across the sampling sites, with several locations showing no detectable levels (ND). Region-2 and Region-2 and Region-7 exhibited the highest LADD for BDCM and DBCM, though these values were still significantly lower than those for CF. Bromoform (BF) was not detected at any of the sites. The cumulative LADD for all THMs was highest in Region-7, reflecting the elevated levels of CF, while the average total LADD for all sampling sites during winter was 2.128 μg/kg/day. These results highlight the importance of continuous monitoring and assessment of THM exposure, particularly in areas like Region-7 where levels are significantly elevated.

Seasonal and annual variations in the Life Average Daily Dose (LADD) of total trihalomethanes (THMs) for Alexandria was shown in Table 5. The data indicates that chloroform (CF) has the highest LADD among the THMs, with values of 1.127 μg/kg/day in summer and 1.929 μg/kg/day in winter, leading to an annual average of 1.5265 μg/kg/day. Bromodichloromethane (BDCM) and dibromochloromethane (DBCM) showed lower LADD values, with BDCM recording 0.307 μg/kg/day in summer and 0.101 μg/kg/day in winter, and DBCM showing 0.173 μg/kg/day in summer and 0.098 μg/kg/day in winter. Bromoform (BF) was not detected (ND) in either season. The total LADD is higher in winter (2.128 μg/kg/day) than in summer (1.607 μg/kg/day), resulting in an annual average of 1.8675 μg/kg/day. This seasonal variation suggests that THM exposure is higher in winter, which could be due to changes in water treatment processes or environmental conditions. The findings highlight the need for seasonal adjustments in monitoring and managing THM levels to minimize health risks.

**Table 5:** Total THMs Life Average Daily Dose variations in summer and winter seasons (μg/kg day).

THM	Summer	Winter	Annual
CF	1.127	1.929	1.5265
BDCM	0.307	0.101	0.204
DBCM	0.173	0.098	0.271
BF	ND	ND	ND
Total THM	1.607	2.128	1.8675

*Cf* chloroform, *BDCM* bromodichloromethane, *DBCM* dibromochloromethane, *BF* bromoform, *ND* not detected.

### The hazard quotient (HQ) and the hazard index (HI) of THM

The hazard quotient (HQ) was calculated based on the comparison of actual exposure to the reference dose (RfD). The HQ estimations for ingestion, dermal, and inhalation were considered for a water consumption rate of 2 L /day. The highest HQ value was CF and the lowest HQ value was BF during all seasons, so CF had more risk in health impacts from other components. During summer, by oral pathway, the HQ_CF_ value ranged between 0–0.405, the HQ_BDCM_ values ranged between 0–0.027, the HQ_DBCM_ ranged between 0–0.01, and the HQ_BF_ values were neglected. For the dermal pathway, the HQ_CF_ value ranged between 0–0.0052, the HQ_BDCM_ values ranged between 0–0.0034, the HQ_DBCM_ ranged between 0–0.0011, and the HQ_BF_ values were neglected for all sites in Alexandria destination during the summer season.

Chloroform (HQCF) presents the greatest hazard, especially in Region-2(HI = 0.443), followed by Region-6 and Region-7. Bromodichloromethane (HQBDCM) also shows a significant risk, particularly in Region-2, while dibromochloromethane (HQDBCM) poses a lower risk but is still notable in Region-2 and Region-6. Bromoform (HQBF) was not detected, indicating no risk from this compound. Overall, the HQs for these THMs are below the critical threshold of 1, indicating a relatively low risk of non-carcinogenic effects. Areas with higher concentrations, like Region-2, warrant continued monitoring to ensure public safety. The estimated non-carcinogenic risk (Hazard Quotient, HQ) was calculated for trihalomethanes (THMs) during the winter season in various locations of Alexandria. Chloroform (HQCF) presents the most significant risk, especially in Region-7 (HI = 0.5698) and Region-4 (HI = 0.1139), with both oral and dermal exposures contributing to the hazard. Bromodichloromethane (HQBDCM) and dibromochloromethane (HQDBCM) show lower risks, with notable levels in Region-2 and Region-5, though still well below the critical threshold of 1. Bromoform (HQBF) was not detected in any location. Overall, while the HQs indicate a low risk of non-carcinogenic effects, the elevated levels in Region-7 suggest a need for closer monitoring and possible intervention to reduce exposure.

The results indicate significant seasonal variations in risk levels. In the summer, Region-2 exhibits the highest cumulative HI (ƩHI) of 0.4843, driven predominantly by chloroform (HICF = 0.443), although this value remains below the critical threshold of 1, suggesting a generally low risk. The high summer HI in Region-2 can be attributed to higher THM concentrations and increased water usage during warmer months, which may elevate exposure levels. In contrast, the winter data show a notable increase in cumulative HI at Region-7 (0.5952), largely due to elevated chloroform levels (HICF = 0.5698). This rise in risk during winter could be linked to changes in water treatment practices or reduced water turnover rates, leading to higher THM concentrations in the distribution network. The need for targeted risk management strategies that account for seasonal fluctuations in THM levels and exposure, ensuring effective public health protection throughout the year is highly recommended^27,28^.

The Total Hazard Index (ƩHI) for trihalomethanes (THMs) levels across various districts in Alexandria. Table 6 shows a critical need for attention to seasonal variations in non-carcinogenic risk. The summer values indicate that Region-2 has the highest ƩHI at 0.4843, reflecting significant exposure risk due to elevated THM concentrations. In winter, Region-7 presents a substantially increased ƩHI of 0.5952, highlighting it as the highest-risk area during this season. This marked seasonal variation suggests that environmental factors and water treatment processes may fluctuate throughout the year, influencing THM levels and subsequently, risk levels. The Total Hazard Index of 2.0034 for all pathways across districts signifies a considerable overall risk from THMs, emphasizing the urgent need for continuous monitoring and improved water management practices. These results justify the necessity for targeted interventions and regulatory measures to mitigate THM exposure and protect public health effectively^29^.

**Table 6:** Estimated non-carcinogenic risk (Total Hazard Index) of THMs levels for random districts located in Alexandria.

Variables	ƩHI		Total
	Summer	Winter	
Region-1	0.0473	0.0126	0.0599
Region-2	0.4843	0.1528	0.6371
Region-3	0.1176	ND	0.1176
Region-4	0.0443	0.1218	0.1661
Region-5	ND	0.215	0.215
Region-6	0.081	0.0726	0.1536
Region-7	0.0589	0.5952	0.6541
Total Hazard Index of all pathway from all pathways			2.0034

### Carcinogenic risks for THM

#### Multi-pathway evaluations of lifetime cancer risks for THM

The exposure metric used for carcinogenic risk assessment is the Lifetime Average Daily Dose (LADD). The average lifetime cancer risk posed by four THMs (CF, BDCM, DBCM, and BF) via three exposure routes is shown in Table 7. The highest cancer risk was in winter. CF contributed to the highest cancer risk from other THMs, and BF contributed to the lowest cancer risk. Ingestion was found to be the most prominent exposure pathway followed by dermal absorption and inhalation. The highest cancer risk was for CF as 53.495 × 10^–6^ and the lowest was for DBCM as 6.27 × 10^–4^ in winter season. The cancer risk of BF in all districts in the different studied seasons was not detected. The results showed that the lifetime cancer risk for all THM components is 67.027 × 10^–6^ which was higher than the USEPA range of concern limit of 1.0 × 10^–6^^30^, about 67 times.

**Table 7:** The lifetime cancer risk posed by all THMs via the three exposure routes at Alexandria districts during summer and winter seasons.

THM	Type of exposure	Cancer risk (10^–6^)							
		The lifetime cancer risk during summer							
		Region-1	Region-2	Region-3	Region-4	Region-5	Region-6	Region-7	Average
CF	Oral	9.92	125.5	26.04	7.13	ND	13.33	10.54	27.49
	Dermal	1.519	23.62	3.22	1.519	ND	2.85	2.29	5.002
	Inhalation	0.0022	0.027	0.0047	0.0023	ND	0.0042	0.0034	0.0062
	**Total**	**11.44**	**149.14**	**29.26**	**8.651**	**ND**	**16.18**	**12.83**	**32.498**
BDCM	Oral	14.26	33.48	19.22	12.4	ND	22.94	16.12	16.92
	Dermal	1.798	4.278	2.418	1.55	ND	2.914	2.046	2.143
	Inhalation	ND	ND	ND	ND	ND	ND	ND	ND
	**Total**	**16.058**	**37.758**	**21.638**	**13.95**	**ND**	**25.854**	**18.166**	**19.063**
DBCM	Oral	14.28	16.8	16.8	11.76	ND	19.32	14.28	13.32
	Dermal	1.26	1.512	1.512	1.092	ND	1.764	1.26	1.2
	Inhalation	ND	ND	ND	ND	ND	ND	ND	ND
	**Total**	**15.54**	**18.312**	**18.312**	**12.852**	**ND**	**21.084**	**15.54**	**14.52**
BF	Oral	ND	ND	ND	ND	ND	ND	ND	ND
	Dermal	ND	ND	ND	ND	ND	ND	ND	ND
	Inhalation	ND	ND	ND	ND	ND	ND	ND	ND
	**Total**	**ND**	**ND**	**ND**	**ND**	**ND**	**ND**	**ND**	**ND**
The lifetime cancer during winter									
CF	Oral	3.534	38.75	ND	31.89	55.8	18.6	159.43	44.001
	Dermal	0.74	8.37	ND	6.85	11.99	3.999	34.41	9.48
	Inhalation	0.0011	0.0123	ND	0.0101	0.0175	0.0059	0.0503	0.0139
	**Total**	**4.275**	**47.132**	**ND**	**38.75**	**67.808**	**22.605**	**193.89**	**53.495**
BDCM	Oral	ND	8.866	ND	ND	8.866	ND	21.266	5.571
	Dermal	ND	1.116	ND	ND	1.116	ND	2.666	0.699
	Inhalation	ND	ND	ND	ND	ND	ND	ND	ND
	**Total**	**ND**	**9.982**	**ND**	**ND**	**9.982**	**ND**	**23.932**	**6.27**
DBCM	Oral	ND	9.576	ND	12.012	12.012	9.576	9.576	7.536
	Dermal	ND	0.84	ND	1.092	1.092	0.84	0.84	0.672
	Inhalation	ND	ND	ND	ND	ND	ND	ND	ND
	**Total**	**ND**	**10.416**	**ND**	**13.104**	**13.104**	**10.416**	**10.416**	**8.208**
BF	Oral	ND	ND	ND	ND	ND	ND	ND	ND
	Dermal	ND	ND	ND	ND	ND	ND	ND	ND
	Inhalation	ND	ND	ND	ND	ND	ND	ND	ND
	**Total**	**ND**	**ND**	**ND**	**ND**	**ND**	**ND**	**ND**	**ND**

These results agree with the study of Semerjian and Dennis^13^ and Amjad, Hashmi^31^. These higher values of cancer risk may cause several diseases among the exposed population. In another study by Siddique, Saied^32^, it was reported the human health cancer risk for TTHMs through ingestion and dermal routes were estimated in "acceptable-low risk" (≥ 1.0 × 10^–6^; ≤ 5.10 × 10^–5^) whereas through inhalation route it was estimated under "acceptable-high risk" (≥ 5.10 × 10^–5^; ≤ 1.0 × 10^–4^) category^32^. Tables 7 provide insights into the estimated lifetime cancer risk from trihalomethanes (THMs) through various exposure routes across Alexandria districts during summer and winter seasons. The data highlight substantial variability in cancer risk associated with different THMs and seasonal changes. During the summer, Chloroform (CF) exhibits the highest cancer risk, with total risks ranging from 8.65 × 10^–6^ in Region-4 to 149.14 × 10^–6^ in Region-2, and an average of 32.50 × 10^–6^. This elevated risk is primarily due to high oral exposure levels in Region-2 and Region-1, reflecting increased THM concentrations and frequent water usage patterns in these areas. Bromodichloromethane (BDCM) also contributes significantly, with total risks from 13.95 × 10^–6^ to 37.76 × 10^–6^ and an average of 19.06 × 10^–6^, driven by oral and dermal exposure. The cancer risk from Dibromochloromethane (DBCM) is lower, ranging from 12.85 × 10^–6^ to 18.31 × 10^–6^, with oral exposure being the major contributor. The absence of Bromoform (BF) detection indicates no risk from this compound, consistent with its lower prevalence in the samples.

In winter, the cancer risk from *Chloroform (CF)* rises significantly, with total risks ranging from 4.275 × 10^–6^ in Region-1 to 193.89 × 10^–6^ in Region-7, averaging 53.50 × 10^–6^. This increase is particularly notable in Region-7 and Region-5, where elevated THM levels and environmental factors such as lower water temperatures may contribute to higher THM concentrations. BDCM and DBCM also contribute to the overall cancer risk, though to a lesser degree compared to CF, with totals from 9.982 × 10^–6^ to 23.932 × 10^–6^ and 10.416 × 10^–6^ to 13.104 × 10^–6^, respectively. The non-detection of Bromoform (BF) in winter samples reinforces its minimal risk profile. These outcomes highlight the need for enhanced water quality monitoring and management strategies in Alexandria, particularly in areas with high THM levels, to mitigate cancer risks associated with THM exposure. The marked seasonal variations also suggest the importance of adapting risk management practices to seasonal changes in water quality^23,33^.

The results from Table 8 emphasize a critical public health concern regarding the lifetime cancer risk posed by trihalomethanes (THMs) in Alexandria. Specifically, Chloroform (CF) is the most significant contributor to cancer risk, with summer and winter values of 32.498 × 10^–6^ and 53.495 × 10^–6^, respectively, leading to an annual average risk of 42.9965 × 10^–6^. This substantial risk increase during winter may be attributed to higher levels of chlorination and disinfection byproducts, which are more concentrated in colder months due to changes in water treatment practices and temperature-dependent chemical reactions.

**Table 8:** The average lifetime cancer risk posed by THMs via the three exposure routes.

Variables	Type of exposure	Cancer risk (10^–6^) Season		Annual (10^–6^)
		Summer	Winter	
CF	Oral	27.49	44.001	
	Dermal	5.002	9.48	
	Inhalation	0.0062	0.0139	
	**Total**	**32.498**	**53.495**	**42.9965**
BDCM	Oral	16.92	5.571	
	Dermal	2.143	0.699	
	Inhalation	ND	ND	
	**Total**	**19.063**	**6.27**	**12.6665**
DBCM	Oral	13.32	7.536	
	Dermal	1.2	0.672	
	Inhalation	ND	ND	
	**Total**	**14.52**	**8.208**	**11.364**
BF	Oral	ND	ND	
	Dermal	ND	ND	
	Inhalation	ND	ND	
	**Total**	**ND**	**ND**	**ND**
TTHM	**Total**	**66.081**	**67.973**	**67.027**

Bromodichloromethane (BDCM) and dibromochloromethane (DBCM) also contribute notably to cancer risks, though their levels are lower compared to CF. BDCM’s total cancer risk is 19.063 × 10^–6^ in summer and decreases to 6.27 × 10^–6^ in winter, reflecting variability in exposure due to seasonal changes in water distribution and treatment processes. Similarly, DBCM shows a total risk of 14.52 × 10^–6^ during summer and 8.208 × 10^–6^ during winter. The reduction in risk for both BDCM and DBCM in winter suggests potential improvements or changes in water treatment or a seasonal dilution effect. Bromoform (BF) was not detected in the samples, which indicates that its contribution to cancer risk in Alexandria is negligible. The total average cancer risks for summer and winter are 66.081 × 10^–6^ and 67.973 × 10^–6^, respectively, with an annual average of 67.027 × 10^−6^. This overall high risk emphasizes the need for continuous monitoring and improvement of water treatment processes to mitigate the presence of THMs and protect public health^34,35^.

Previous research in European countries such as Italy, Lithuania, Spain, and the United Kingdom has found THM levels comparable to those observed in this study. However, THM levels were lower in Greece (specifically Crete) and higher in France (Rennes region) than the national averages reported here^36,37^. Outside the European Union, THM concentrations have varied widely: from 6.2 μg/L in Dharan, Saudi Arabia (2012)^38^ to 21.1 μg/L in Tetovo, North Macedonia (2011)^39,40^, 35.4 μg/L in Ankara, Turkey (2016), 43.9 μg/L in Quebec, Canada (2000–2001)^41^, and 260 μg/L in Islamabad, Pakistan (2012)^31^.

Over the past two decades, many European Union countries have successfully reduced THM levels in public drinking water through various improvements in treatment methods and infrastructure. Advances include optimizing disinfection processes and enhancing water resource management^42–45^. For example, in France, efforts have been made to decrease soluble organic matter in surface water sources and optimize chlorine dosage to minimize disinfection byproduct (DBP) formation^46,47^. Italy, meanwhile, utilizes chlorine dioxide extensively, which lowers THM levels but increases chlorite and chlorate concentrations^48^. Other countries employ ozone (e.g., the Netherlands, Germany, France), UV radiation (Austria), or chloramines (e.g., Finland, Sweden) to achieve lower THM levels.

Nevertheless, each disinfection method generates different DBPs, such as aldehydes, ketones, carboxylic acids (from ozonation), bromate (from ozonation in the presence of bromide), nitrosamines (from chlorination), and chlorite/chlorate (from chlorine dioxide)^49–52^. These disinfectants are highly reactive and lead to the formation of various DBPs, many of which are not regulated and may be carcinogenic or genotoxic, with potential links to bladder cancer^53^. THMs are often used as indicators of total DBP content in epidemiological studies, but they have limitations as markers since they do not represent the full range of DBPs and are not always the most toxic^54^. Their effects are complex due to interactions with other DBPs, and correlations with specific DBPs can vary^55,56^. Despite these limitations, THMs remain a valuable measure for assessing overall DBP exposure. The current investigation involves statistical and cancer risk analysis of THM species in Alexandria’s drinking water, highlighting the importance of such assessments in informing public health policies and ensuring water safety. Quantifying these risks is crucial for effective decision-making regarding drinking water safety.

## Conclusions and recommendations

This study investigated the seasonal and spatial variations of trihalomethanes (THMs) in Alexandria’s drinking water and assessed their associated non-carcinogenic and carcinogenic health risks through ingestion, dermal, and inhalation exposure routes. The results demonstrated that THM levels vary significantly between regions and seasons, with the highest concentrations detected in Region-2 during the summer (150 µg/L) and in Region-7 during the winter (196 µg/L). While elevated temperatures in summer generally promote THM formation due to enhanced reaction kinetics, this pattern was not consistent across all districts. The observed increase in THMs during the winter in certain areas may be attributed to site-specific factors such as water stagnation, infrastructure condition, or disinfection practices. Critical districts, such as Region-2 and Region-7, exhibit elevated cancer risk values, highlighting the urgent need for targeted water treatment improvements. While some areas meet international standards, others exceed recommended safety limits, necessitating the adoption of advanced treatment technologies like ozone and UV disinfection. Optimizing chlorine dosing, enhancing distribution infrastructure, and implementing a robust monitoring system are crucial for long-term water safety.

However, this study has certain limitations. Key water quality parameters, such as pH, residual chlorine, and total organic matter, were not measured, which may have provided further insights into THM formation dynamics. Additionally, the study focused on specific regions within Alexandria, and broader investigations covering multiple water sources and treatment plants would offer a more comprehensive understanding. Future research should explore the mechanistic pathways of THM formation and assess alternative disinfection methods to mitigate their occurrence. Additionally, investigating the impact of water source variability and emerging contaminants will contribute to more adaptive and sustainable water management strategies. Public awareness, regulatory updates, and investment in research will further support sustainable water quality management and improved public health outcomes.

## Data Availability

The datasets generated and analyzed during the current study are available from the corresponding author upon reasonable request.

## Abbreviations

THMs: Trihalomethanes
USEPA: United States Environmental Protection Agency
CF: Chloroform
IWHO: World Health Organization Index for Additive Toxicity
HQ: Hazard quotient
LADD: Life average dily dose
HI: Hazard Index
BDCM: Bromodichloromethane
BF: Bromoform
DBCM: Dibromochloromethane
GV: Guideline value
WHO: World Health Organization
RfD: Reference dose
MCL: Maximum contaminant level
CSF: Cancer slope factor
DBPs: Disinfection by-products
EPA: Environmental protection agency
